# Cyclodextrin-Based Quercetin Powders for Potential Nose-to-Brain Transport: Formulation and In Vitro Assessment

**DOI:** 10.3390/molecules30132878

**Published:** 2025-07-07

**Authors:** Elmina-Marina Saitani, Paraskevi Papakyriakopoulou, Theodora Bogri, Georgia Choleva, Kyriaki Kontopoulou, Spyridon Roboras, Maria Samiou, Antiopi Vardaxi, Stergios Pispas, Georgia Valsami, Natassa Pippa

**Affiliations:** 1 Section of Pharmaceutical Technology, Department of Pharmacy, School of Health Sciences, National and Kapodistrian University of Athens, Panepistimiopolis Zografou, 15771 Athens, Greece; e.saitani@pharm.uoa.gr (E.-M.S.); theodorabgr@pharm.uoa.gr (T.B.); geocholeva@gmail.com (G.C.); kyriakicont@gmail.com (K.K.); roborasspy@gmail.com (S.R.); msamiou@pharm.uoa.gr (M.S.); avardaxi@eie.gr (G.V.); natpippa@pharm.uoa.gr (N.P.); 2 Theoretical and Physical Chemistry Institute, National Hellenic Research Foundation, 48 Vassileos Constantinou Avenue, 11635 Athens, Greece; valsami@pharm.uoa.gr (A.V.); pispas@eie.gr (S.P.)

**Keywords:** quercetin, cyclodextrins, nasal powders, freeze-drying, spray-drying, thermogravimetric analysis, scanning electron microscopy, in vitro diffusion experiments

## Abstract

Quercetin (Que) is widely recognized for its antioxidant and neuroprotective properties; however, its clinical potential remains limited due to poor solubility and low oral bioavailability. Nasal powders have emerged as a promising strategy to overcome these limitations, taking advantage of nose-to-brain delivery, offering a direct, non-invasive route to the central nervous system while bypassing first-pass metabolism. This study aims to extend previous work by systematically investigating the impact of different preparation methods (spray drying vs. lyophilization) and the incorporation of hydroxypropyl methylcellulose (HPMC) and mannitol/lecithin microparticles (MLMPs) on the physicochemical characteristics, structural properties, and in vitro diffusion behavior of HPβCD-based nasal powder formulations of Que. Thermal behavior and stability were analyzed using TGA, while morphology and particle distribution were assessed via Scanning Electron Microscopy. In vitro diffusion studies using Franz cells and regenerated cellulose membranes were conducted under simulated nasal conditions. Among all tested formulations, the spray-dried HPβCD/Que powder (F4) showed the highest permeation (0.11 ± 0.01 mg/cm^2^ at 120 min). The inclusion of HPMC improved thermal stability but reduced Que diffusion, likely due to increased viscosity and matrix formation. Blending with MLMPs enhanced powder flow and dose placement, although it modestly reduced diffusion efficiency. Overall, this study highlights the potential of HPβCD-based spray-dried powders for nasal Que delivery and demonstrates how HPMC and MLMPs can be strategically employed to tailor performance characteristics.

## 1. Introduction

Quercetin (Que), a flavonoid found widely in the plant kingdom [1], exhibits important pharmacological properties, including antioxidant, anti-inflammatory, anticancer, antidiabetic, and antimicrobial effects [2,3,4,5]. It has gained significant research interest, particularly for its potential in preventing and treating diseases like cancer, cardiovascular conditions, and age-related disorders, like Alzheimer’s disease (AD) [1,6,7]. It is involved in multiple molecular activities, including the inhibition of β-amyloid aggregation, the reduction of oxidative stress, the modulation of neuroinflammation, and mitochondrial protection [8]. These effects are largely attributed to its ability to regulate key signaling pathways, such as PI3K/Akt, JAK/STAT, and NF-κB, which are involved in neuronal survival, antioxidant defense, and inflammation control [9,10]. In AD and aging models, quercetin has been shown to restore cognitive function, inhibit tau phosphorylation, and reduce amyloid burden, indicating its potential as a multitarget compound for the prevention and treatment of AD [11,12]. Despite these promising findings, the clinical application of quercetin remains limited due to its poor bioavailability and insufficient brain penetration. In particular, when Que is administered orally, it presents poor bioavailability due to its low solubility and extensive first-pass metabolism, which significantly limit its therapeutic application [13].

Currently, most drugs available on the market for managing Alzheimer’s disease are administered orally, intravenously, or intramuscularly. However, achieving effective drug concentrations in the bloodstream faces multiple challenges. Hepatic first-pass metabolism, enzymatic degradation in the gastrointestinal tract, and the restrictive nature of the BBB substantially limit systemic bioavailability and hinder the timely delivery of the active pharmaceutical ingredient (API) to the CNS [14]. These challenges highlight the need for alternative administration routes that can bypass metabolic and anatomical barriers to improve brain targeting. Among these, the intranasal route offers distinct advantages over oral administration, addressing some of its challenges [15]. The direct neuronal connection between the olfactory region and the CNS, bypassing the BBB and avoiding first-pass metabolism, facilitates nose-to-brain API delivery. Thus, this non-invasive and patient-friendly administration approach allows for more precise drug targeting, higher concentrations in the brain, and an improved safety profile [15].

Recent nose-to-brain delivery strategies for quercetin include liquid or semi-liquid formulations such as intranasal transferosomal and niosomal gels (≈170–200 nm), which have demonstrated sustained release, enhanced nasal permeability, and effective brain targeting in rodent models [16]. However, these systems often involve complex lipid compositions, are prone to physical instability, and may cause mucosal irritation. Additionally, polymeric nanocapsules (~228 nm) have shown improved anxiolytic efficacy compared to oral administration but are limited by an initial burst release and variability in dose delivery [17]. Although liquid formulations are commonly used in nasal drug delivery, they are generally more suitable for local or topical effects rather than systemic or brain targeting due to several limitations, such as the risk of formulation leakage from the nasal cavity, dose variability, unintentional swallowing, and gastrointestinal absorption [14]. In contrast, nasal powders offer several key benefits as they exhibit higher physicochemical stability, prolonged residence time in the nasal cavity, and improved mucoadhesion [18]. These features enable higher local drug concentrations at the nasal mucosa, which can translate into increased bioavailability [19] and improved CNS targeting, making nasal powders particularly advantageous for the management of CNS disorders [20,21].

To fully harness the advantages of nasal powder formulations, appropriate drying techniques are essential for producing stable, solid-state products. Among the various methods used to remove water from pharmaceutical materials, freeze-drying is a well-established technique to produce lyophilized products in the pharmaceutical industry. It offers minimal process losses and straightforward aseptic processing. Through the lyophilization process, the risk of biomolecule denaturation is minimized due to the low temperatures used [22,23]. This technique can be combined with spray-drying processes that offer greater control over particle characteristics, such as size, morphology, and surface properties, which are attributes considered to be critical in nasal drug delivery. It is also well-suited to continuous manufacturing and enables the customization of particles for targeted delivery routes. While spray-drying can suffer from higher process losses compared to freeze-drying, these can be mitigated through process optimization. The technique also involves a complex interplay of parameters, requiring careful and case-by-case, time-intensive fine-tuning. Both methods present distinct advantages and challenges, and their suitability depends on the specific formulation goals and processing constraints [22,23].

In our previous work, we investigated lyophilized compositions of Que with methyl-β-cyclodextrin (MβCD) and hydroxypropyl-β-cyclodextrin (HPβCD) (Que-MβCD and Que-HPβCD, respectively) [24], as well as nasal powders of Que-CDs lyophilizates blended with spray-dried mannitol/lecithin microparticles (MLMPs) [25]. These formulations were evaluated through in vitro diffusion experiments and ex vivo permeation studies using the rabbit nasal mucosa. Building on these findings, a pharmacokinetic study was conducted in Wistar rats to compare intranasal versus oral administration of Que lyophilizates and their blends with MLMPs (75:25 *w*/*w*) [26]. The results demonstrated the promising potential of these nasal powders for both systemic and nose-to-brain delivery in the prevention and/or management of neuroinflammatory degenerative disorders, such as Parkinson’s and Alzheimer’s disease. Notably, HPβCD increased the water solubility of pure Que by approximately 50-fold, outperforming the respective MβCD/Que formulation, which achieved a 40-fold increase. In line with this, the pharmacokinetic analysis revealed that the HPβCD/Que lyophilizate produced the highest serum bioavailability, while its blend with the MLMPs showed the most efficient brain targeting among all the tested formulations.

Based on the findings of our previous research, we selected the HPβCD/Que lyophilized formulation for further investigation due to its superior solubility enhancement and bioavailability profile. The aim of this study was to explore how the co-incorporation of mannitol and lecithin into the same freeze-drying batch might impact the formulation’s in vitro release profile [HPβCD/Que/mannitol/lecithin freeze-dried (F1)]. Additionally, we investigated the potential role of the widely used hydrophilic carrier HPMC, a hydrophilic polymer widely used in nasal sprays and mucoadhesive systems [27,28,29], in modulating the formulation’s behavior by incorporating it either alone [HPMC/HPβCD/Que freeze-dried (F2)] or in combination with mannitol and lecithin [HPMC/HPβCD/Que/mannitol/lecithin freeze-dried (F3)]. In parallel, we assessed whether spray-drying could produce powders with better in vitro release profiles compared to lyophilization. For this reason, two spray-dried formulations were prepared: HPβCD/Que (F4) and HPMC/HPβCD/Que (F5). Finally, to evaluate the effect of MLMPs on the spray-dried systems, F4 and F5 were blended with spray-dried MLMPs in three different weight ratios: 25:75, 50:50, and 75:25 (F6–F8 and F9–F11, respectively). Scanning Electron Microscopy (SEM) analysis was employed to assess the surface roughness and morphology of both the individual powders and the blended systems. All formulations, including both freeze-dried and spray-dried powders, were further characterized via in vitro release studies to investigate potential excipient interactions, the influence of HPMC and MLMPs on powder characteristics, and the contribution of MLMPs to the morphological and biopharmaceutical properties relevant to nasal delivery.

## 2. Results and Discussion

### 2.1. Yield of Spray-Drying Process

The % yield of the spray-drying process was calculated as the ratio of the mass of recovered microparticles (MPs) to the initial mass of total solids in the feed solution. Table 1 summarizes the % yields obtained across all spray-drying experiments. The production of spray-dried MLMPs resulted in a yield of 26.0%, using the procedure described in Section 3.2.2. Notably, formulation F4 achieved a yield of 41.0%, more than double that of formulation F5, which yielded only 20.5%.

### 2.2. Que Content in the Powders and Blends

In the current study, three lyophilized powders (F1–F3) and two spray-dried formulations (F4 and F5) were produced. The spray-drying powders F4 and F5 were used for the preparation of blends with MLMPs in three different ratios (formulation/MLMPs 25:75, 50:50, and 75:25) (Section 3.2.5). Table 2 shows the values of the theoretical Que content (%), as well as the experimental Que content (%) and the calculated recovery (%). The data represents the mean ± SD of three replicates. The Que content in the freeze-dried and spray-dried powders ranged from 1.87 ± 0.04% to 7.92 ± 0.29% (*w*/*w*), whereas in the blends of the spray-dried formulations with MLMPs, it varied from 1.44 ± 0.01% to 5.87 ± 0.14% (*w*/*w*) (Table 2). The recovery values, calculated as the percentage of the detected amount of Que relative to the theoretical one, varied from 68.61 ± 1.69% to 119.14 ± 0.48% (Table 2). These results indicate that the recovery of powders from the lyophilization process (especially for the formulations F2 and F3, which contained HPMC) was lower than that achieved through the spray-drying process.

### 2.3. Decoding System’s Thermal Stability

**Figure 1:**
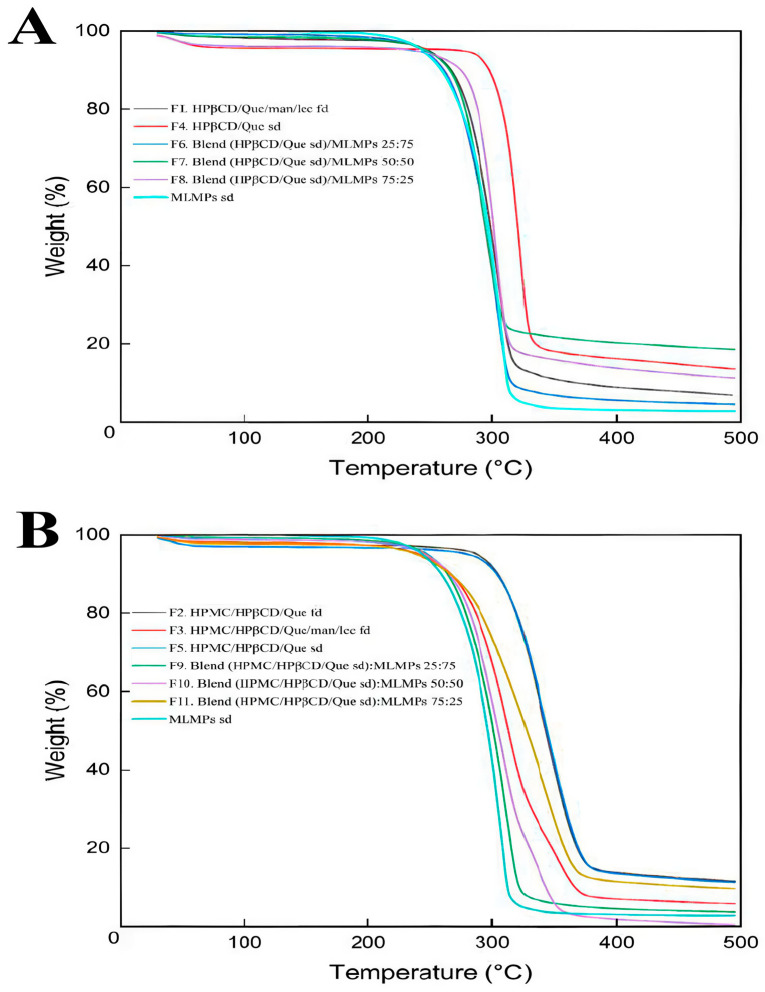
TGA curves for (**A**) HPβCD/Que/mannitol/lecithin freeze-dried (F1), Que/HPβCD spray-dried (F4), blends of Que/HPβCD with MLMPs at three different weight ratios (F6–F8), and MLMPs spray-dried and (**B**) HPMC/HPβCD/Que freeze-dried (F2), HPMC/HPβCD/Que/mannitol/lecithin freeze-dried (F3), HPMC/HPβCD/Que spray-dried (F5), blends of HPMC/HPβCD/Que spray-dried with MLMPs at three different weight ratios (F9–F11), and MLMPs spray-dried. The axes denote the mass change (%) vs. temperature (°C).

The degradation process of the prepared formulations is depicted in Figure 1. In the literature, it was reported that the TGA analysis of pure Que revealed three stages of degradation: water loss (6.5%) at 94–137 °C, significant mass loss (27.3%) at 240–385 °C, and further decomposition (38%) at 386–900 °C, leaving 28.2% residue at 900 °C [30]. Based on Figure 1, it can be concluded that HPβCD and/or HPMC positively influenced Que’s thermal stability as the onset temperature of the main thermal event occurred beyond 200 °C in all the tested formulations.

TGA was used in order to investigate the thermal stability of both the freeze- and spray-dried prepared systems. Zhu et al. [31] described the degradation of HPβCD via TGA as a multi-phase process. Initially, there is a slight weight loss due to the evaporation of water. This is followed by a significant weight loss during the primary pyrolysis phase, occurring between 309.5 °C and 420 °C. Finally, the last phase involves the decomposition of HPβCD, which occurs between 420 °C and 500 °C. Notably, other studies also reported that the main thermal decomposition of HPβCD occurs within the 300 °C to 400 °C range [32,33]. The enhanced thermal stability observed in the HPβCD-containing systems was due to the movement of the onset of the thermal degradation at higher temperatures, attributed to the encapsulation of Que within the lipophilic cavity of the HPβCD matrix. This inclusion complex stabilized Que by shielding it from thermal stress, while van der Waals interactions between Que and HPβCD further reinforced the complex, increasing its resistance to degradation at elevated temperatures. These findings are consistent with previously reported studies [33]. For all systems, the minor weight loss observed between 30 and 200 °C (Supplementary Materials, Figure S1) was attributed to moisture desorption, consistent with earlier findings [34,35]. Among the formulations without HPMC, F4 (HPβCD/Que sd) demonstrated superior thermal stability, as indicated by its higher onset decomposition temperature and slower decomposition rate compared to F1 and F6–F9 (Figure 1A).

According to the literature, the main decomposition of mannitol is observed via TGA between 280 and 350 °C, where the breaking of the polyol structure takes place [36]. Around the temperature range of 200–250 °C, lecithin is also degraded by breaking the phospholipid structure [37]. This phenomenon is also concentration-dependent, leading to reduced thermal stability of the samples because the decomposition temperature decreases as the number of hydroxyl groups in the samples increases, as in the case of MLPMPs. When comparing TGA results for formulations with the same compositions but prepared using different methods [freeze-drying (F2) vs. spray-drying (F5)], it could be observed that TGA thermograms appeared to be nearly identical. This strongly indicated that the preparation method did not affect the thermal decomposition behavior of the formulations. Formulation F3 contained the additional excipients mannitol and lecithin, compared to F2. The presence of these excipients was associated with a reduction in the onset temperature of degradation. This suggests that mannitol and lecithin may influence thermal behavior by causing the degradation of the system at lower temperatures. The extent of their impact can vary depending on factors such as the manufacturer, formulation process, concentration, and the specific compound they are combined with. Previous studies have also confirmed via TGA that the presence of mannitol or lecithin can degrade the system’s or substance’s thermal stability [38,39]. This study presents, to the best of current knowledge, the first use of TGA to investigate how different preparation methods influence the thermal stability of compositionally identical formulations.

The MLMPs sd formulation (cyan curve, Figure 1) had the lowest onset and decomposition temperatures among the samples, indicating that MLMPs alone are less thermally stable compared to HPβCD/Que or HPMC/HPβCD/Que complexes (~210 °C). This suggests that MLMPs contributed to the early degradation of the formulation. Hence, their inclusion in the composition of F4 formulation negatively impacted its thermal stability, as evidenced by the lower degradation onset in MLMPs containing formulations (F6, F7, and F8) compared to the pure complex in F4 (Figure 1A). A similar pattern was observed in HPMC-based systems, where the formulation without MLMPs (F5) demonstrated higher thermal stability than the corresponding MLMP-containing blends (F9–F11).

According to previous studies, pure HPMC undergoes a single-stage thermal degradation, reaching its maximum degradation temperature at 393.5 °C, with a mass loss of 77.3%. The main degradation process takes place between roughly 320 °C and 420 °C [30]. The TGA results showed that adding HPMC to the HPβCD/Que system improved thermal resistance and protected the material from earlier decomposition (Figure 1B). This improvement was attributed to the formation of additional hydrogen bonds/interactions between HPMC, HPβCD, and Que, which enhanced the stability of the complex structure and, in turn, reduced its decomposition. Indeed, the presence of HPMC in F5 likely provided additional thermal protection, as demonstrated by higher onset and decomposition temperatures, as well as slightly lower weight loss compared to F4. The enhanced thermal stability offered by HPMC was also confirmed by Guo et al. (2022), who demonstrated that adding HPMC to κ-carrageenan films not only enhanced thermal stability but also improved mechanical properties and barrier functions, owing to increased hydrogen bonding and a more compact film structure [40].

Furthermore, formulations with HPMC (F9, F10, F11) demonstrated better thermal stability compared to their counterparts without HPMC (F6, F7, F8), as indicated by higher onset degradation temperatures and lower overall weight loss. Among these, F11 (HPMC/HPβCD/Que sd–MLMPs 75:25) showed the best thermal stability, with the lowest weight loss and a higher resistance to decomposition. This suggests that increasing the proportion of MLMPs compromised the thermal resistance of the blend. The one-step degradation of HPβCD/Que sd–MLMPs blends (F9, F10, F11) occurred between 240 °C and 320 °C. Notably, F10, which contained a 50:50 ratio of the complex to MLMPs, underwent complete degradation by the end of the experiment, further highlighting the destabilizing effect of a higher MLMP content.

Overall, these findings underscore the critical role of formulation composition in determining thermal stability. Additionally, the inclusion of HPMC appears to provide significant protection against thermal degradation, likely through hydrogen bonding and structural stabilization. Conversely, the presence of MLMPs adversely affects thermal resistance. Importantly, the observed degradation temperatures remained well above physiological conditions and are therefore not relevant for in vivo medical applications [41].

### 2.4. Morphological Characterization of Que Lyophilized and Spray-Dried Powders and Blends

SEM was chosen to evaluate directly the shape, surface morphology, and possible existence of agglomeration phenomena. SEM is an ideal method for the determination of the morphology of non-spherical and irregularly shaped particles [42]. On the other hand, this technique allows for the visualization of particles in a solid state and offers a quantitative evaluation of samples directly (not using volume-based/number-based statistics or the assumption of a spherical shape using the Stokes–Einstein equation for a hydrodynamic diameter) [43]. Particle size and morphology are critical quality attributes for achieving the effective nose-to-brain delivery of powder formulations as they significantly influence deposition in the olfactory region and subsequent drug transport to the brain. Ideally, microparticles intended for this route should range between 5 and 50 or 100 µm to optimize nasal deposition while minimizing clearance by mucociliary action and enhancing uptake through the olfactory and trigeminal pathways [44,45]. To assess the suitability of the prepared formulations, SEM analysis was employed to examine their morphology and determine whether the particle sizes fell within the optimal range for brain targeting via nasal administration. Additionally, the effects of different preparation methods (freeze-drying and spray-drying) and the presence of various excipients on particle size and morphology were also investigated, as these factors can significantly impact the aerodynamic properties and the stability of the final powder [46].

**Figure 2:**
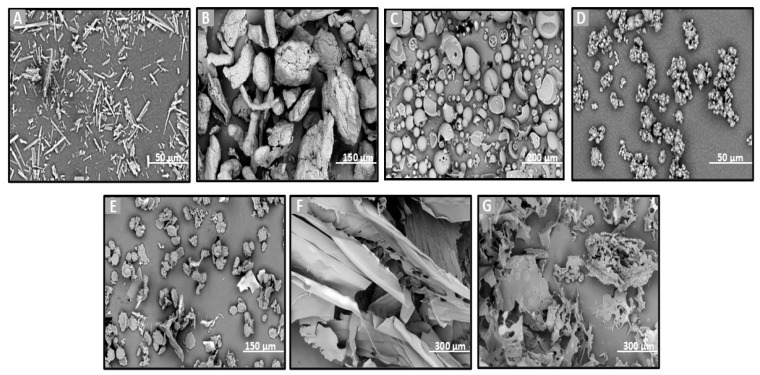
SEM images of (**A**) Que, (**B**) HPMC, (**C**) HPβCD, (**D**) MLMP, and F1–F3 lyophilized powders (**E**–**G**). (**A**,**D**) at ×3000 magnification, (**B**,**E**) at ×1000 magnification, (**C**) at ×600 magnification, (**F**,**G**) at ×500 magnification, and (**B**) at ×560 magnification.

**Figure 3:**
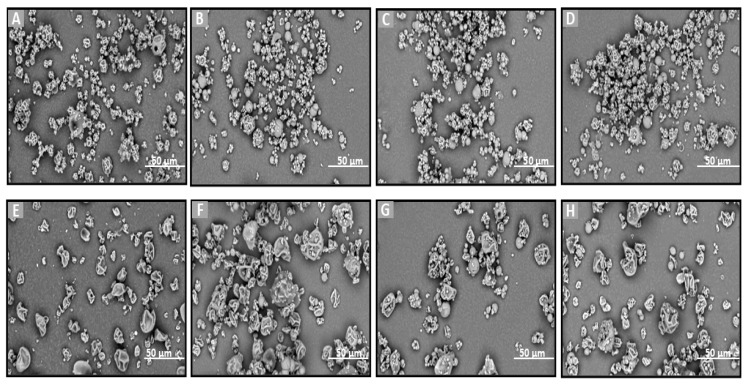
SEM images of (**A**) F4, (**B**–**D**) blends of F4 with MLMPs in three different ratios (formulation/MLMPs 25:75, 50:50, and 75:25) (F6–F8), (**E**) F5, and (**F**–**H**) blends of F5 with MLMPs in three different ratios (formulation/MLMPs 25:75, 50:50, and 75:25) (F9–F11) at ×3000 magnification.

SEM analysis was performed on the raw materials (Que, HPβCD, HPMC, and MLMPs), the lyophilized formulations F1–F3 (Figure 2), the spray-dried powders F4–F5, and the blends of spray-dried formulations with MLMPs in varying weight ratios (F6–F11) (Figure 3). The SEM image of the commercially available Que (Figure 2A) predominantly displayed needle-shaped or rod-shaped forms of various sizes, characteristic of a crystalline morphology [25]. However, other morphological forms—such as irregular and rough surfaces—have also been observed in pure Que, depending on the manufacturer [47]. HPMC (Figure 2B) appeared as highly porous, irregularly shaped agglomerates with coarse, rough surfaces. The morphology of pure HPβCD (Figure 2B) was in agreement with previous findings reported in the literature [25,48], showing mainly spherical structures with hollow cores.

The lyophilization process favored the formation of amorphous solids due to the sublimation of water molecules from the frozen solutions [25]. This effect is clearly observable in the SEM micrographs of the freeze-dried formulations F1–F3 (Figure 2E–G). The SEM analysis revealed that all lyophilized powders, whether containing HPMC (F2 and F3 formulations) or not (F1 formulation), exhibited morphologies markedly distinct from those of the raw materials. Specifically, the lyophilized products appeared as flake-like particles with irregular shapes and a broad size distribution.

The incorporation of MLMPs in nasal powder formulations is well-documented in the literature [25,49]. SEM analysis confirmed that MLMPs exhibited a predominantly spherical morphology with a marked tendency to form agglomerates (Figure 2D). The impact of different preparation methods on powder morphology was subsequently evaluated by comparing spray-dried and freeze-dried samples [specifically, HPβCD/Que spray-dried (F4) versus freeze-dried [25] and HPMC/HPβCD/Que spray-dried (F5) vs. freeze-dried (F2)]. As expected, lyophilization tended to produce more amorphous solids relative to their spray-dried counterparts. Furthermore, blending the lyophilized powders with MLMPs in various weight ratios for 20 min significantly altered their morphology compared to the unblended formulations. This was particularly evident in the transitions from F4 to F6–F8 and from F5 to F9–F11, leading to the formation of agglomerates (Figure 3). The MLMPs appeared to cover the surfaces of the spray-dried complex particles (HPβCD/Que sd or HPMC/HPβCD/Que), with smaller MLMPs adhering to the larger host particles. Increasing the MLMP content led to more extensive surface coverage, suggesting the potential for enhanced mucoadhesive interaction and the modulation of aerodynamic behavior.

In all tested formulations, the powders exhibited a dominant population of fine particles smaller than 30 μm, with most particles measuring under 10 μm in diameter. Regarding the formulations F4 and F5, more than 80% of the particle population fell below 10 μm, while approximately 20% ranged between 10 and 30 μm. The incorporation of MLMPs and the resulting formation of powder blends promoted agglomeration, as MLMPs adhered to the surfaces of the unblended formulation particles. Interestingly, although agglomerates were formed, the predominant particle population in the blends shifted toward smaller sizes with increasing MLMP content (Table 3), suggesting a redistribution effect induced by surface coating and blending dynamics. The distinct morphological features observed via SEM analysis were found to correlate closely with the drug release performance of the Que formulations. Specifically, the spray-dried formulations produced spherical particles with relatively smooth surfaces and narrow size distributions. These morphological traits are advantageous for nasal delivery as they enhance powder dispersibility, increase the surface area available for wetting, and promote more rapid interaction with the dissolution medium, thus facilitating drug release [50]. In contrast, the lyophilized powders (F1–F3) exhibited irregular, flake-like structures with broader size distributions, potentially limiting surface exposure and reducing diffusion efficiency. This morphological disparity was consistent with the significantly higher quercetin permeation observed for spray-dried F4 (0.11 ± 0.01 mg/cm^2^ at 120 min) compared to the lyophilized counterparts.

The development of MPs for Que delivery was previously reported in the literature. For instance, Rosita et al. (2022) developed a solid lipid microparticle system for the pulmonary delivery of Que, using glyceryl behenate as the lipid matrix and Poloxamer 188 as the surfactant [51]. The MPs were prepared via freeze-drying and exhibited a spherical shape with smooth surfaces and sizes ranging from 1.8 to 1.9 μm. Li et al. (2014) [52] prepared fast-dissolving core–shell composite MPs of Que using coaxial electrospraying, resulting in particles with clear core–shell architectures and diameters between 1.3 and 1.7 μm. However, the particles obtained in those studies were considerably smaller and exhibited distinct morphologies compared to those in the present work. These differences likely stemmed from variations in formulation components, preparation methods, and drying techniques, all of which play a pivotal role in determining the final particle characteristics.

### 2.5. Que’s Release from the Lyophilized and Spray-Dried Powders and Blends

**Figure 4:**
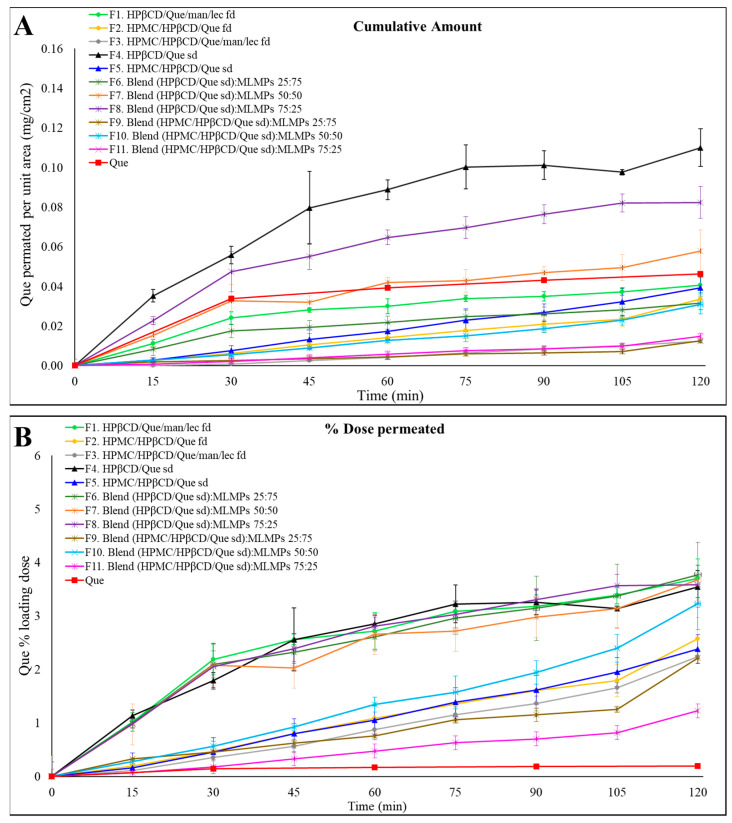
Permeation profiles of the prepared formulations (F1–F11) through regenerated cellulose membranes compared to Que solution. The results are expressed as (**A**) the quantity permeated per unit area (mean ± SD, n = 3) and (**B**) the percentage (%) of the loading dose permeated for the tested formulation (mean ± SD, n = 3).

To evaluate the in vitro release and diffusion behavior of Que from the freeze-dried and spray-dried powders, as well as the blends of spray-dried powders with varying amounts of MLMPs, Que diffusion was examined using Franz cells and a regenerated cellulose membrane serving as a diffusion barrier. The 1000 Da MW cutoff of the membrane permitted the free Que to flow through while preventing the permeation of excipients. The mass balance results encompass the total Que permeated per unit area (mg/cm^2^), the total (%) permeated amount, the calculated Que quantity in the donor compartment, and the Que amount remaining in the membrane at the end of the experiment (Table 4). Figure 4 presents the results of the in vitro release experiments conducted with the prepared formulations. Using a consistent powder amount led to varying Que loading doses in the donor compartment across formulations. Therefore, the total permeated Que is also expressed as a percentage of the loading dose to facilitate comparisons between formulations.

Notably, F4 (HPβCD/Que sd) demonstrated the highest permeation levels, expressed as the amount of Que permeated per unit area, reaching 0.11 ± 0.01 mg/cm^2^ at 120 min, followed by its blend with 25% MLMPs (F8). These in vitro findings align well with our earlier observations. In our previous study [25], we demonstrated a strong correlation between in vitro diffusion profiles through regenerated cellulose membranes and ex vivo release results, with formulations showing the same relative ranking in terms of quercetin permeated per unit area. Based on this validated relationship, ex vivo experiments were not repeated in the present work, as the in vitro model was deemed sufficient for a comparative evaluation of formulation performance at this stage. Furthermore, prior in vivo studies confirmed the nose-to-brain delivery potential of the Que–HP-β-CD lyophilized formulation; however, handling limitations were associated with its low-density, voluminous texture [26]. In the current study, we employed spray-drying to prepare the HP-β-CD complex. This modification not only enhanced the formulation’s rheological properties but was also expected to improve its in vivo performance. To further support its suitability for nasal delivery, we also included an in vitro sprayability test of F4 and F8 compared to the freeze-dried counterparts, using a nasal spray device adapted for rodent administration [Section 2.6].

The presence of MLMPs decreased the amount of Que permeated per unit area at all tested ratios (Figure 4A, Table 4) compared to the pure lyophilized powder [F4 > F6–F8, *p* > 0.05, 95% Confidence Interval (CI)] (Supplementary Materials, Figure S2) and F5 > F9 (*p* > 0.05, 95% CI), F10, and F11 (*p* > 0.05, 95% CI)] (Supplementary Materials, Figure S3). This result was expected as a reduction in the amount of Que correspondingly decreased the available surface area for diffusion. Additionally, it lowered the likelihood that the entire powder would be efficiently wetted when 100 μL of buffer (pH = 5.6) was added to the donor compartment.

The comparable or even higher permeation observed for formulations F1–F3, F5–F7, and F9–F11 relative to pure Que (Figure 4A) could be attributed to the significantly higher loading dose of pure Que (15 mg), which was 9 to 42 times greater than that of the other formulations. In contrast, the loading doses for all tested formulations ranged from 0.36 to 1.98 mg (Table 4). Notably, formulations F4 and F8 exhibited 2.40-fold and 1.77-fold higher amounts of Que permeated per unit area, respectively, compared to the pure Que, although these differences were not statistically significant (*p* > 0.05, 95% CI). Regarding the impact of HPMC on Que permeation through cellulose membranes, the data in Figure 4A show that, within formulations prepared using the same technique, the inclusion of HPMC in the formulation composition led to a reduced amount of Que permeated per unit area compared to their HPMC-free counterparts (e.g., F1 vs. F2 and F4 vs. F5), although these differences were not statistically significant (*p* > 0.05, 95% CI). The most pronounced difference was observed between the spray-dried formulations, where F4 achieved approximately three times greater Que permeation per unit area than F5 by the end of the experiment. Notably, F4 demonstrated the highest permeation levels across all time points, reaching 0.11 ± 0.01 mg/cm^2^ at 120 min. Additionally, the incorporation of MLMPs resulted in a decrease in Que permeated per unit area at all tested ratios when compared to the pure lyophilized powder.

Other studies reported in the literature also investigated the use of MPs as drug delivery systems of Que. The MPs that were fabricated by Rosita et al. [42] were composed of a solid lipid and a surfactant, with varying lipid concentrations. In vitro release studies were conducted in phosphate buffer solution (PBS) at pH 7.4 and 37 °C, using a 15 mg Que loading dose. The results demonstrated a sustained release profile for Que, with approximately 30% of the Que released over 10.5 h [51]. Another study reported [52] another approach based on fast-dissolving core–shell MP delivery systems using polyvinylpyrrolidone, sodium dodecyl sulfate, and sucralose. The systems were fabricated through coaxial electro-spraying and intended for the oral or sublingual delivery of Que. The results of in vitro permeation studies (PBS pH 6.8, 37 °C) revealed that the MPs rapidly released the incorporated Que within one minute and presented a 10-fold faster permeation rate across the sublingual mucosa than pure API [45]. A study by Scalia et al. (2013) [53] reported the fabrication of controlled-release inhalable lipid MPs loaded with Que, employing various lipid materials and surfactants. In vitro release studies were performed in PBS (0.05 M, pH 7.4) at 37 °C, supplemented with 0.5% (*w*/*w*) polysorbate 20. Among the formulations tested, lipid MPs composed of tristearin and hydrogenated phosphatidylcholine demonstrated the most efficient modulation of Que release, exhibiting sustained release characteristics [53]. Unlike lipid-based MPs, where drug solubilization occurs within a hydrophobic core requiring surfactant-mediated release, the hydrophilic matrix in the HPMC/CD-based system enabled the direct dissolution of Que, contributing to a potential rapid delivery to the brain, as also proved with CD-based systems [26].

However, direct comparisons with the present study’s results are not feasible due to variations in experimental conditions and the materials used. Furthermore, the in vitro experiments differed in terms of Que loading doses, duration, and the implementation of the in vitro experiment techniques. Notably, in vitro release studies with artificial membranes showed that the presence of HPMC caused a retarding effect on the diffusion of Que, which is also supported in the literature. In particular, Jaipal et al. [54] utilized a factorial design to prepare buccal discs of buspirone hydrochloride using HPMC and mannitol. They evaluated the impact of polymer concentration and mannitol levels on the drug release profile. Their findings revealed that increasing the concentration of HPMC reduced the drug release rate, while higher levels of mannitol had the opposite effect. This may be attributed to HPMC’s impact on increasing viscosity and extending its diffusional path length, leading to a more pronounced delay in buspirone release. Overall, the retarding HPMC’s impact on increasing viscosity, forming a film on the membrane surface, or potential interactions between the hydrophilic polymer and cyclodextrins, such as hydrophobic interactions or hydrogen bonds, alters the system’s physicochemical properties. Notably, the presence of sugars has been reported to suppress the water interaction with HPMC polymer chains, thereby slowing gel layer formation while promoting the rapid development of an effective diffusion barrier. This phenomenon has been observed even at low HPMC concentrations, such as 1% [55]. The viscosity-modulating properties of HPMC, along with its potential interactions with the natural sugars commonly used in drug formulations, can be strategically utilized for tailoring drug release rates in hydrophilic matrices.

### 2.6. In Vitro Device-Based Assessment of Powder Emission

An in vitro test was performed to evaluate the delivery performance of F4 and F8, which demonstrated the best diffusion profile (Figure 4A), as well as the corresponding freeze-dried formulation assessed in our previous work [25]. Formulation F4 (HPβCD/Que spray-dried) showed a mean emitted powder dose of 7.26 ± 1.57 mg (n = 5) (Table 5), indicating excellent dispersibility and consistent emission. In contrast, formulation F8 (HPβCD/Que spray-dried, 75:25 ratio) yielded a significantly lower emitted dose of 1.43 ± 0.38 mg (n = 5) (Table 5). The freeze-dried version of F4 (HPβCD/Que freeze-dried) resulted in a zero emitted dose under the same test conditions, indicating that the flake shape of lyophilized powder does not allow powder release through this device. On the other hand, the freeze-dried counterpart of F8 (a 75:25 blend of HPβCD/Que and MLMPs) achieved an emitted dose approximately twice as high as the spray-dried F8 formulation, suggesting that the freeze-drying process may have favorably altered the powder’s physical properties to improve aerosolization in the blended formulations. These findings highlight the crucial influence of formulation composition and processing method on delivery performance. While spray-dried F4 demonstrates strong potential for intranasal delivery, the performance of F8-type powders (blend with MLMPs) remains suboptimal and requires further optimization to achieve reliable and efficient administration.

## 3. Materials and Methods

### 3.1. Chemicals and Reagents

Que (MW: 302.24 g/mol), MβCD (MW: 1310 g/mol), and HPβCD (MW: 1460 g/mol) were purchased from Sigma-Aldrich (St. Louis, MO, USA), FlukaChemika (Mexico City, Mexico), and Ashland (Covington, KY, USA), respectively. HPMC (Methocel E50 premium LV, MW: 90.000 g/mol) was purchased from Colorcon (Shanghai, China). Ethanol HPLC-grade was purchased from Fischer Scientific (Pittsburgh, PA, USA). Mannitol (Ph. Eur.) was supplied by Fagron (Glinde, Germany) and lecithin from soybean was supplied by Santa Cruz Biotechnology (Dallas, TX, USA). Regenerated cellulose membranes (MW cut-off 1000 Da, diameter 63 nm) were obtained from Spectrum Laboratories (Gardena, CA, USA). Triple-deionized water from Millipore was used for all preparations.

### 3.2. Methods

#### 3.2.1. Preparation of Freeze-Dried Formulations

Regarding the preparation of spray-dried Que/HPβCD/mannitol/lecithin powders, the Que/HPβCD complex was formed at a molar ratio of 1:2. A mannitol/lecithin mixture (92:8 *w*/*w*) was also added to achieve a weight ratio of (Que/HPβCD)/(mannitol/lecithin) equal to 50:50. First, the required amount of mannitol was transferred to a beaker with water under continuous stirring. An ethanolic lecithin solution (20 mg/mL) was then added, followed by the addition of HPβCD. Afterwards, Que was added under continuous stirring and light protection (due to Que’s photosensitivity) and the formation of the suspension was observed. Small amounts of a 6% ammonium hydroxide solution were gradually added to ensure complete Que dissolution, with the pH adjusted to approximately 9 during this process. To carry out the lyophilization process, the obtained colloidal dispersions were dispensed into circular trays, frozen at −73 °C, and freeze-dried using the Biobase Vacuum Freeze-Dryer, BK-FD10T, Biobasebiodustry CO., LTD (Jinan, China). The content of RH in the resulting lyophilized powders was subsequently quantified using high-performance liquid chromatography (HPLC) analysis with a photodiode array detector (PDA) [Section 3.2.6.].

#### 3.2.2. Preparation of Spray-Dried Formulations

##### Preparation of Spray-Dried Que/HPβCD Powder

The spray-dried formulation of Que/HPβCD was produced by spray-drying the liquid solution of Que/HPβCD using the neutralization method [56], with a molar ratio of 1:2. More specifically, the required amount of HPβCD was weighed and suspended in water until complete CD solubilization. The subsequent addition of Que and the adjustment of pH were performed as previously described.

The feed solution was spray-dried using a Mini Spray-Dryer B-191 (BÜCHI Labortechnik AG, Flawil, Switzerland) with an inlet temperature set at 100 °C, a flow rate of 5.7 mL/min, aspiration at 100%, and airflow maintained at 600 NL h^−1^. After the spray-drying process, the resulting powders were stored in dark-colored containers. The yield from the spray-drying process was calculated by dividing the mass of the recovered MPs by the initial mass of total solids in the feed solution.

##### Preparation of Spray-Dried HPMC/HPβCD/Que Powder

Afterwards, it was also decided to manufacture the spray-dried products with the addition of HPMC 1%. For this reason, the required amount of HPβCD was added to a beaker containing water under stirring. The solution was then heated to 75–80 °C. Once the desired temperature was reached, the heating process was stopped, and HPMC was gradually added to the solution. When the mixture became uniformly cloudy, the beaker was transferred to a refrigerator to facilitate gel formation. After the gel formed, it was stirred using a magnetic stirrer until its uniform solubilization. The subsequent steps, including the addition of Que, pH adjustment, and spray-drying conditions, were carried out as described in Section 3.2.1 and Section 3.2.2.

##### Preparation of Spray-Dried MLMP Powder

For the preparation of spray-dried MLMPs, a feed solution was produced by combining an aqueous mannitol solution with an ethanolic solution of lecithin, achieving a weight ratio of the components of 92:8. Following the procedure established by Balducci et al. [57], the experimental conditions for the spray-dryer were a liquid feed rate of 6.5 mL/min, with the inlet temperature set to 100 °C, aspiration at 100%, and airflow maintained at 600 NL h^−1^.

#### 3.2.3. Preparation of Blends

Blends comprising spray-dried MLMPs with the spray-dried Que/HPβCD and HPMC/HPβCD/Que (F3–F5 and F7–F9, respectively) were manually prepared in glass vials using a spatula, with varying ratios [formulation/MLMPs 25:75 (blend A), 50:50 (blend B), and 75:25 (blend C)]. Each mixture was stirred for 20 min, following the protocol described by Papakyriakopoulou et al. (2021) [25]. These specific ratios were selected based on our previous work [25], which demonstrated their suitability for evaluating the full spectrum of MLMP-related effects on formulation synthesis, including blend uniformity, flowability, and powder performance. The quantification of Que in each formulation was conducted through HPLC analysis following the conditions described in Section 3.2.6. Table 6 presents all the formulations prepared by spray-drying and freeze-drying processes.

#### 3.2.4. Thermogravimetric Analysis

Thermogravimetric analysis (TGA) was conducted using the TA instrument Q500 TGA Analyzer in the temperature range of 30–500 °C at a heating rate of 10 °C/min in an air atmosphere [58].

#### 3.2.5. Scanning Electron Microscopy (SEM) Analysis 

SEM analysis was conducted to investigate the effects of the preparation techniques and the contribution of each material to the morphology of the final formulations. The raw material powders (Que, HPβCD, HPMC, and MLMPs), the freeze-dried and spray-dried powders (F1–F3 and F4–F5, respectively), and the blends of the spray-dried powders with MLMPs (F6–F11) were examined using a Phenom ProX G6 desktop scanning electron microscope (Thermo Fischer Scientific, Waltham, MA, USA) and a charge reduction sample holder. The samples were inspected at a 10 kV accelerating voltage, without sputter coating, and the particle size distribution was assessed using ImageJ 1.54 software by manually measuring the dimensions of the agglomerates or particles’ sides (at least 100 individual particles per sample across multiple fields of view) and calculating their average size.

#### 3.2.6. Quantitative Analysis of Que

The Que content in the prepared systems and the samples from the in vitro experiments was quantified using HPLC-PDA following appropriate dilutions, based on a previously validated method [25]. The analysis was conducted on a Shimadzu prominence system (Kyoto, Japan) equipped with an LC-20AD Quaternary Gradient Pump with a degasser, a SIL-HT auto-sampler, and a SPD-M20A PDA detector. Data acquisition and nd processing were carried out using LC solution^®^ software (LabSolutions, version 1.25 SP4, Kyoto, Japan). Chromatographic separation was achieved using a reverse-phase Thermo Aquasil C18 column (150 × 4.6 mm, 5 μm) protected by a matching C18 guard column (12.5 × 4.6 mm, 5 µm particle size). The mobile phase consisted of a mixture of methanol/water (65:35 *v*/*v*) and the pH value was adjusted to 2.8 with orthophosphoric acid. The flow rate was maintained at 1.0 mL/min and the injection volume was set at 20 µL. Detection was performed at a wavelength of 369 nm (λmax). A calibration curve was constructed using a methanolic stock solution of Que (1 mg/mL) and dilutions in the mobile phase, covering a concentration range of 1 to 40 µg/mL.

#### 3.2.7. In Vitro Diffusion Experiments

In vitro diffusion experiments were performed using regenerated cellulose membranes with a molecular cut-off of 1000 Da (Spectra/Por^®^ 7 Dialysis Membrane, Pretreated RC Dialysis Tubing, Spectrum Laboratories, Inc., Gardena, CA, USA) mounted on Franz-type diffusion cells (Crown Glass, Somerville, MA, USA).

Membrane preparation involved a 15 min soak in distilled water, followed by a rinse with fresh distilled water and a second immersion for 30 min. The membranes were then equilibrated in PBS (pH adjusted to 7.4) for 15 min. After pre-treatment, the membranes were cut into 1 cm^2^ squares to fully cover the diffusion area of the Franz cells (0.636 cm^2^). For the diffusion cell set up, 5 mL of PBS was added to the receptor compartment, and the pre-treated membrane was placed between the donor and receptor chambers. The compartments were then clamped securely. The entire system was allowed to equilibrate at 34 °C to mimic the temperature of the nasal cavity.

Then, 25 mg of each formulation (Table 7) or 15 mg of pure lyophilized Que were placed in the donor compartment and wet with 100 μL of a buffer with a pH of 5.6. The Que dose ranged from 0.36 to 1.98 mg, resulting in donor concentrations between 3.6 and 19.8 mg/mL. These values exceeded the solubility threshold reported in our previous studies, indicating that the donor compartment was saturated under all conditions [59]. The donor and receptor compartments were both covered with Parafilm^®^ to prevent evaporation and a magnetic stirrer was added in the receptor compartment.

The diffusion area (*A*) of the Franz cell was equal to 0.636 cm^2^. The flux (*J*) across the artificial membrane from the donor to the receptor compartment was calculated using the slopes obtained by regression analysis of the amount of Que (*Q*) permeated per unit area (*A*) vs. the time, as shown in Equation (1) [60].

(1) $$J=\frac{dQ}{dt\times A}$$

#### 3.2.8. In Vitro Device-Based Assessment of Powder Emission

The in vitro performance of four powder formulations was evaluated using a modified Dry Powder Penwu device to assess emitted dose consistency and efficiency. The Dry Powder Penwu device was adapted for murine intranasal administration. In this modified version, the upper steel tube was replaced with the plastic upper part of a 22G IV catheter to better fit the nasal anatomy of mice. This adaptation was motivated by the fact that most in vivo models for neurodegenerative diseases are based on mice rather than rats, owing to the broader availability of genetically modified murine models and their well-characterized behavioral and pathological features [61]. Each formulation was weighed and loaded into the device at a target dose of 10 mg. The emitted dose was assessed by drawing 1.5 mL of air through the device using a 5 mL syringe, simulating the actuation conditions described for in vivo use. The emitted dose for each formulation was collected on a pre-weighed filter and quantified by determining the weight difference before and after actuation. Emitted dose was expressed as a percentage of the initial loaded dose. The test was performed five times for each formulation to ensure reproducibility and the results were reported as the mean ± standard deviation.

#### 3.2.9. Statistical Analysis

Statistical analysis was carried out using the GraphPad Prism 8.0 software package (GraphPad Software, version 8.01). A significance threshold of *p* < 0.05 was applied for all the analyses, with two-tailed tests and a 95% confidence interval (CI). Outlier detection was performed using the interquartile range (IQR) method with a threshold of 1.5 × IQR; no outliers were identified. Comparisons among groups were evaluated using one-way ANOVA, followed by Bonferroni post hoc tests to assess statistically significant differences between all pairs of formulations. All experiments were conducted in triplicate and the results are reported as the mean ± SD. Statistical comparisons of permeation values were performed both across different formulations and at individual time points within each formulation.

## 4. Conclusions

In the present study, we thought it was worth investigating the combination of the diverse materials (HPMC, HPβCD, mannitol, lecithin) for the possible nasal administration of Que. This is the first report to comprehensively compare freeze-dried and spray-dried powders comprising these components, focusing on their thermal behavior, structural characteristics, and in vitro diffusion performance. TGA analysis confirmed that formulation composition critically affected thermal stability. Notably, the incorporation of HPMC provided significant protection against thermal degradation, while the inclusion of MLMPs reduced thermal resilience. Among the tested formulations, F4 (HPβCD/Que spray-dried) exhibited the highest diffusion efficiency, achieving a cumulative permeation of 0.11 ± 0.01 mg/cm^2^ by the end of the experiment (120 min). The addition of MLMPs consistently reduced quercetin permeation across all tested ratios, despite improving handling characteristics and dose placement uniformity. Furthermore, formulations containing HPMC exhibited a retarding effect on Que diffusion, likely due to viscosity-related and barrier-forming properties. When dose-normalized, the results confirmed that F4 also had one of the highest percentages of permeated dose, emphasizing its superior performance in both absolute and relative terms. Given that the F4 formulation exhibited the highest emitted dose and superior dispersibility in vitro—and considering that the corresponding freeze-dried formulation has previously demonstrated the successful delivery of Que to both serum and the brain in vivo—it is reasonable to expect that F4 will also perform effectively under in vivo conditions, potentially offering enhanced therapeutic benefit. Overall, these findings support the potential of HPβCD-based spray-dried powders as promising candidates for nasal Que delivery, though in vivo studies are needed to confirm their nose-to-brain transport capability, as well as any potential therapeutic efficacy.

## Figures and Tables

**Table 1:** % Yield and powder mass (g) of F4, F5, and MLMP formulations produced by spray-drying process.

Formulations	Mass (g) of Produced Powder	Yield (%)
F4: HPβCD/Que spray-dried	1.1	41.0
F5: HPMC/HPβCD/Que spray-dried	1.1	20.5
MLMPs	2.6	26.0

**Table 2:** Theoretical and experimental Que content (%) and recovery (%) in the prepared powders and blends. The data represents the mean ± SD of three replicates.

Formulations	Theoretical Que Content *w*/*w* (%)	Experimental Que Content (*w*/*w*) ± SD (%)	Recovery ± SD (%)
F1	3.26	2.79 ± 0.04	85.61 ± 1.25
F2	4.84	3.32 ± 0.08	68.61 ± 1.69
F3	2.46	1.87 ± 0.04	76.15 ± 1.66
F4	9.39	7.92 ± 0.29	84.33 ± 3.09
F5	4.84	4.19 ± 0.01	86.63 ± 0.17
F6	2.35	2.12 ± 0.11	90.41 ± 4.54
F7	4.71	4.01 ± 0.08	85.24 ± 1.71
F8	7.04	5.87 ± 0.14	83.37 ± 1.98
F9	1.21	1.44 ± 0.01	119.14 ± 0.48
F10	2.42	2.19 ± 0.06	90.64 ± 2.62
F11	3.63	3.07 ± 0.04	84.72 ± 1.06

**Table 3:** Illustration of the % of total population with different sizes of particles.

Blend/% of Total Population with Specific Size Dimensions	50–100 μm	30–50 μm	10–30 μm	<10 μm
F1	0	9	91	0
F4	1	2	16	81
F5	0	0	17	83
F6	0	1	27	72
F7	0	0	18	82
F8	0	0	10	90
F9	0	0	41	59
F10	0	0	14	86
F11	0	0	7	93
MLMPs	0	2	49	49

**Table 4:** Percentage (%) of Que loading dose permeated for the tested formulations (mean ± SD, n = 3), mass balance (%) of Que in each formulation (mean ± SD, n = 3), and membrane retention data of each formulation expressed as the percentage (%) of the loading dose retained by the cellulose membrane (mean ± SD, n = 3).

Formulation	Que Permeated Per Unit Area (mg/cm^2^)	Permeated Que (% Loading Dose)	% of Que Dose Retained by Cellulose Membrane
F1	0.04 ± 0.00	3.70 ± 0.36	1.51 ± 0.55
F2	0.03 ± 0.01	2.57 ± 0.40	0.86 ± 0.02
F3	0.01 ± 0.00	2.24 ± 0.12	5.66 ± 0.19
F4	0.11 ± 0.01	3.54 ± 0.31	1.19 ± 0.33
F5	0.04 ± 0.01	2.38 ± 0.37	1.89 ± 0.76
F6	0.03 ± 0.01	3.77 ± 0.61	1.54 ± 0.32
F7	0.06 ± 0.01	3.68 ± 0.67	1.04 ± 0.32
F8	0.08 ± 0.01	3.58 ± 0.36	1.20 ± 0.44
F9	0.01 ±0.00	2.21 ± 0.00	1.51 ± 0.07
F10	0.03 ± 0.00	3.23 ± 0.49	1.94 ± 0.56
F11	0.01 ± 0.00	1.23 ± 0.07	0.91 ± 0.10
Que control	0.20 ± 0.00	1.45 ± 0.59	0.05 ± 0.00

**Table 5:** In vitro evaluation of emitted dose from the modified Dry Powder Penwu device. The table summarizes the amount of powder loaded into the device, the emitted powder, and the corresponding emitted Que content per actuation (mean ± SD, n = 5).

Formulation	Loaded Powder (mg)	Emitted Powder (mg)
F4	10.42 ± 0.38	7.26 ± 1.57
F8	10.43 ± 0.33	1.43 ± 0.38
HPβCD/Que freeze-dried	10.35 ± 0.24	0
Blend HPβCD/Que freeze-dried–MLMPs 75:25	10.18 ± 0.29	2.78 ± 0.37

**Table 6:** Composition of all the prepared formulations.

	Formulations
F1	HPβCD/Que/mannitol/lecithin freeze-dried
F2	HPMC/HPβCD/Que freeze-dried
F3	HPMC/HPβCD/Que/mannitol/lecithin freeze-dried
F4	HPβCD/Que spray-dried
F5	HPMC/HPβCD/Que spray-dried
F6	Blend HPβCD/Que spray-dried–MLMPs 25:75
F7	Blend HPβCD/Que spray-dried–MLMPs 50:50
F8	Blend HPβCD/Que spray-dried–MLMPs 75:25
F9	Blend HPMC/HPβCD/Que spray-dried–MLMPs 25:75
F10	Blend HPMC/HPβCD/Que spray-dried–MLMPs 50:50
F11	Blend HPMC/HPβCD/Que spray-dried–MLMPs 75:25

**Table 7:** Que content in 25 mg of each formulation.

Formulation	Formulation–MLMPs	Experimental Que Content	
		% *w*/*w* ± SD	Amount (mg) in 25 mg of Formulation ± SD
F1	100:0	2.79 ± 0.04	0.70 ± 0.01
F2	100:0	3.32 ± 0.08	0.83 ± 0.02
F3	100:0	1.87 ± 0.04	0.47 ± 0.01
F4	100:0	7.92 ± 0.29	1.98 ± 0.07
F5	100:0	4.19 ± 0.01	1.05 ± 0.00
F6	25:75	2.12 ± 0.11	0.53 ± 0.03
F7	50:50	4.01 ± 0.08	1.00 ± 0.02
F8	75:25	5.87 ± 0.14	1.47 ± 0.03
F9	25:75	1.44 ± 0.01	0.36 ± 0.00
F10	50:50	2.19 ± 0.06	0.55 ± 0.02
F11	75:25	3.07 ± 0.04	0.77 ± 0.01

## Data Availability

Data are contained within the article and Supplementary Materials.

## Supplementary Materials

The following supporting information can be downloaded at https://www.mdpi.com/article/10.3390/molecules30132878/s1: Figure S1: TGA curves for (A) HPβCD/Que/mannitol/lecithin freeze-dried (F1), Que/HPβCD spray-dried (F4), and blends of Que/HPβCD with MLMPs at three different weight ratios (F6–F8), MLMPs spray-dried and (B) HPMC/HPβCD/Que freeze-dried (F2), HPMC/HPβCD/Que/mannitol/lecithin freeze-dried (F3), HPMC/HPβCD/Que spray-dried (F5), blends of HPMC/HPβCD/Que spray-dried with MLMPs at three different weight ratios (F9–F11), and MLMPs spray-dried. The axes denote the mass change (%) vs. temperature (°C), with a zoomed-in view provided for the temperature range of 0–300 °C to highlight observed differences.; Figure S2. Permeation profiles of F4 and its blends with MLMPs (F6–F8) in different weight ratios (25:75, 50:50, and 75:25) through regenerated cellulose membranes compared to Que solution. The results are expressed as (A) the quantity permeated per unit area (mean ± SD, n = 3) and (B) the percentage (%) of the loading dose permeated for the tested formulation (mean ± SD, n = 3).; Figure S3. Permeation profiles of F5 and its blends (F9–F11) with MLMPs in different weight ratios (25:75, 50:50, and 75:25) through regenerated cellulose membranes compared to Que solution. The results are expressed as (A) the quantity permeated per unit area (mean ± SD, n = 3) and (B) the percentage (%) of the loading dose permeated for the tested formulation (mean ± SD, n = 3). Figure S4. Distribution histograms of HPβCD/Que/mannitol/lecithin freeze-dried (F1), and MLMPs. The x-axis represents particle size range (μm), and the y-axis indicates particle count (n = number of particles measured). Figure S5. Distribution histograms of Que/HPβCD spray-dried (F4) and its blends (F6–F8) with MLMPs in different weight ratios (25:75, 50:50 and 75:25). The x-axis represents particle size range (μm), and the y-axis indicates particle count (n = number of particles measured). Figure S6. Distribution histograms of HPMC/HPβCD/Que spray-dried (F5).and its blends (F9–F11) with MLMPs in different weight ratios (25:75, 50:50 and 75:25). The x-axis represents particle size range (μm), and the y-axis indicates particle count (n = number of particles measured).

## Abbreviations

The following abbreviations are used in this manuscript:

A	Diffusion area
API	Active pharmaceutical ingredient
BBB	Βlood–brain barrier
CI	Confidence interval
CNS	Central nervous system
HPLC	High-performance liquid chromatography
HPMC	Hydroxy-propyl methyl cellulose
HPβCD	Hydroxy-propyl-β-CD
IQR	Ιnterquartile range
J	Flux
Que	Quercetin
MPs	Microparticles
MLMPs	Microparticles of mannitol/lecithin
MW	Molecular weight
ΜβCD	Methyl-β-CD
PDA	Photodiode array
PBS	Phosphate buffer solution
Que	Quercetin
SD	Standard deviation
SEM	Scanning electron microscopy
TGA	Thermogravimetric analysis
