# Supplementary material for: Cyclodextrin-Based Quercetin Powders for Potential Nose-to-Brain Transport: Formulation and In Vitro Assessment
Source: Molecules. 2025 Jul 7;30(13):2878. doi: 10.3390/molecules30132878 (PMC12251352; doi:10.3390/molecules30132878)
Supplement: Supplementary file 1 [file molecules-30-02878-s001.zip › molecules-3729655-supplementary.pdf]

Supplementary Material

Cyclodextrin-Based Quercetin Powders for potential Nose-to-Brain Transport: Formulation and *In Vitro* Assessment

Elmina-Marina Saitani[1], Paraskevi Papakyriakopoulou[1,*], Theodora Bogri[1,#], Georgia Choleva[1,#], Kyriaki Kontopoulou[1,#], Spyridon Roboras[1,#], Maria Samiou[1,#], Antiopi Vardaxi[3], Stergios Pispas[3], Georgia Valsami[1], Natassa Pippa[1]

[1] Section of Pharmaceutical Technology, Department of Pharmacy, School of Health Sciences, National and Kapodistrian University of Athens, Panepistimiopolis Zografou, 15771 Athens, Greece.; e.saitani@pharm.uoa.gr; ppapakyr@pharm.uoa.gr; theodorabgr@pharm.uoa.gr; geocholeva@gmail.com; kyriakicont@gmail.com; roborasspy@gmail.com; msamiou@pharm.uoa.gr; valsami@pharm.uoa.gr; natpippa@pharm.uoa.gr

[2] Theoretical and Physical Chemistry Institute, National Hellenic Research Foundation, 48 Vassileos Constantinou Avenue, 11635 Athens, Greece.; avardaxi@eie.gr; pispas@eie.gr

*Correspondence: ppapakyr@pharm.uoa.gr

#equal contribution to this work

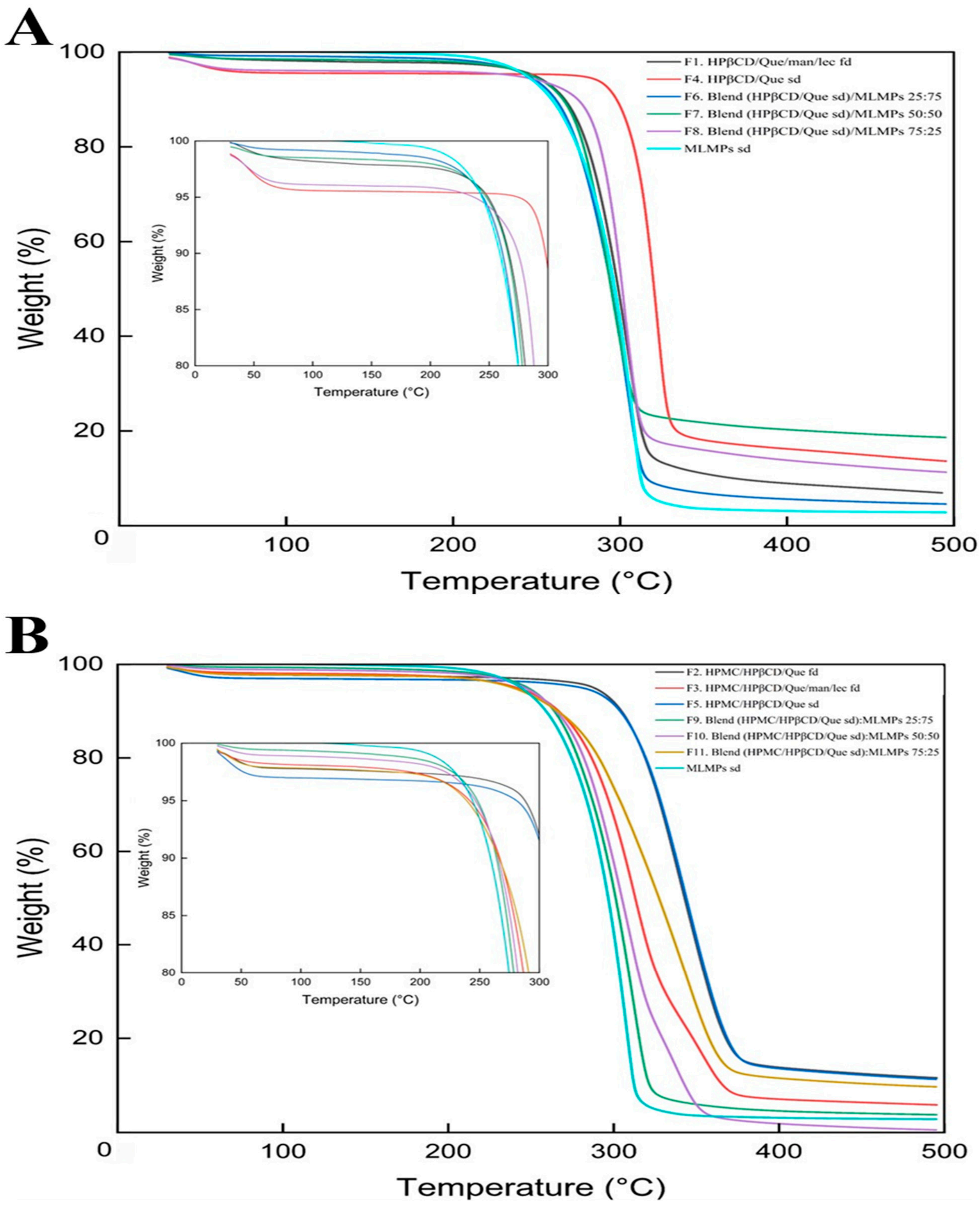

Figure S1. TGA curves for (A) HPβCD/Que/mannitol/lecithin freeze-dried (F1), Que/HPβCD spray-dried (F4), blends of Que/HPβCD with MLMPs at three different weight ratios (F6-F8), MLMPs spray-dried and (B) HPMC/HPβCD/Que freeze-dried (F2) and HPMC/HPβCD/Que/mannitol/lecithin freeze-dried (F3), HPMC/HPβCD/Que spray-dried (F5), blends of HPMC/HPβCD/Que spray-dried with MLMPs at three different weight ratios (F9-F11) and MLMPs spray-dried. The axes denote the mass change (%) vs. temperature (°C), with a zoomed-in view provided for the temperature range of 0-300 °C to highlight observed differences.

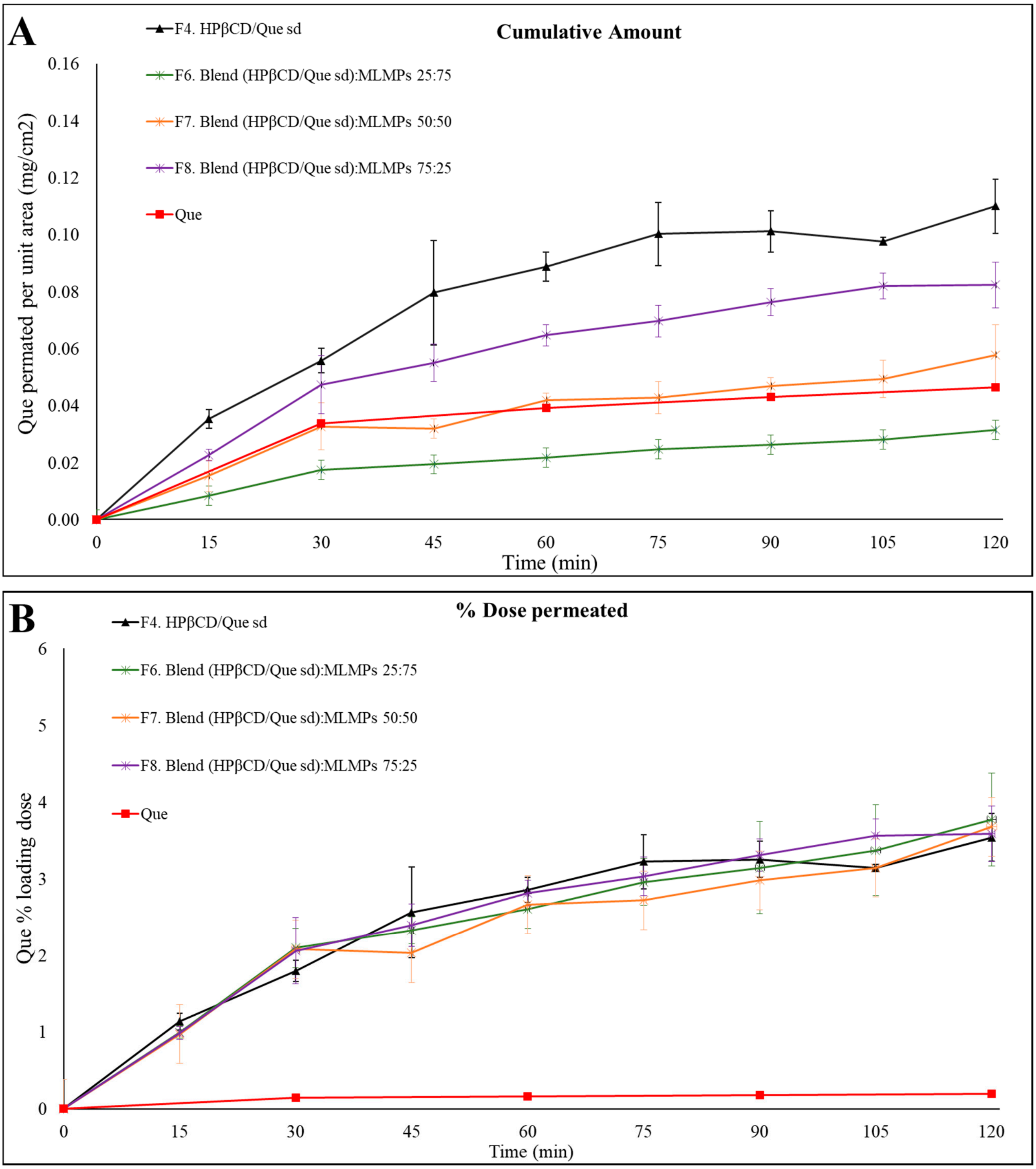

Figure S2. Permeation profiles of the F4 and its blends with MLMPs (F6-F8) in different weight ratios (25:75, 50:50 and 75:25) through regenerated cellulose membranes compared to Que solution. The results are expressed as (A) the quantity permeated per unit area (mean ± SD, n = 3) and (B) the percentage (%) of the loading dose permeated for the tested formulation (mean ± SD, n = 3).

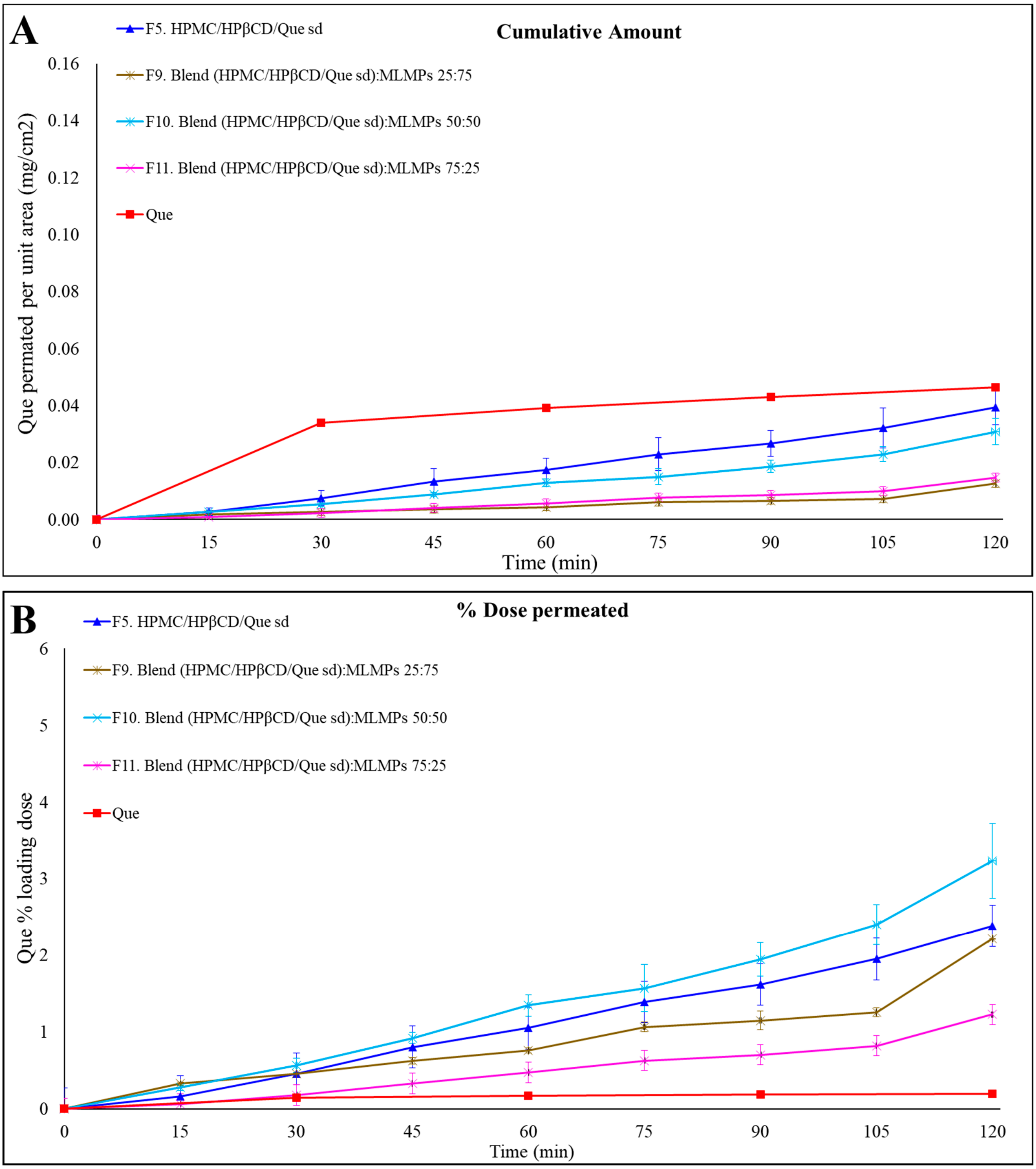

Figure S3. Permeation profiles of the F5 and its blends (F9-F11) with MLMPs in different weight ratios (25:75, 50:50 and 75:25) through regenerated cellulose membranes compared to Que solution. The results are expressed as (A) the quantity permeated per unit area (mean ± SD, n = 3) and (B) the percentage (%) of the loading dose permeated for the tested formulation (mean ± SD, n = 3).

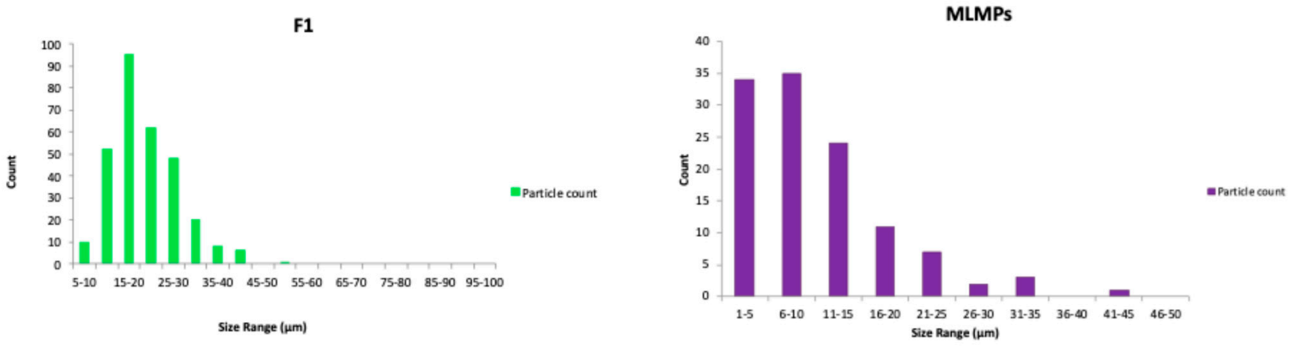

Figure S4. Distribution histograms of HPβCD/Que/mannitol/lecithin freeze-dried (F1), and MLMPs. The x-axis represents particle size range (µm), and the y-axis indicates particle count (n = number of particles measured).

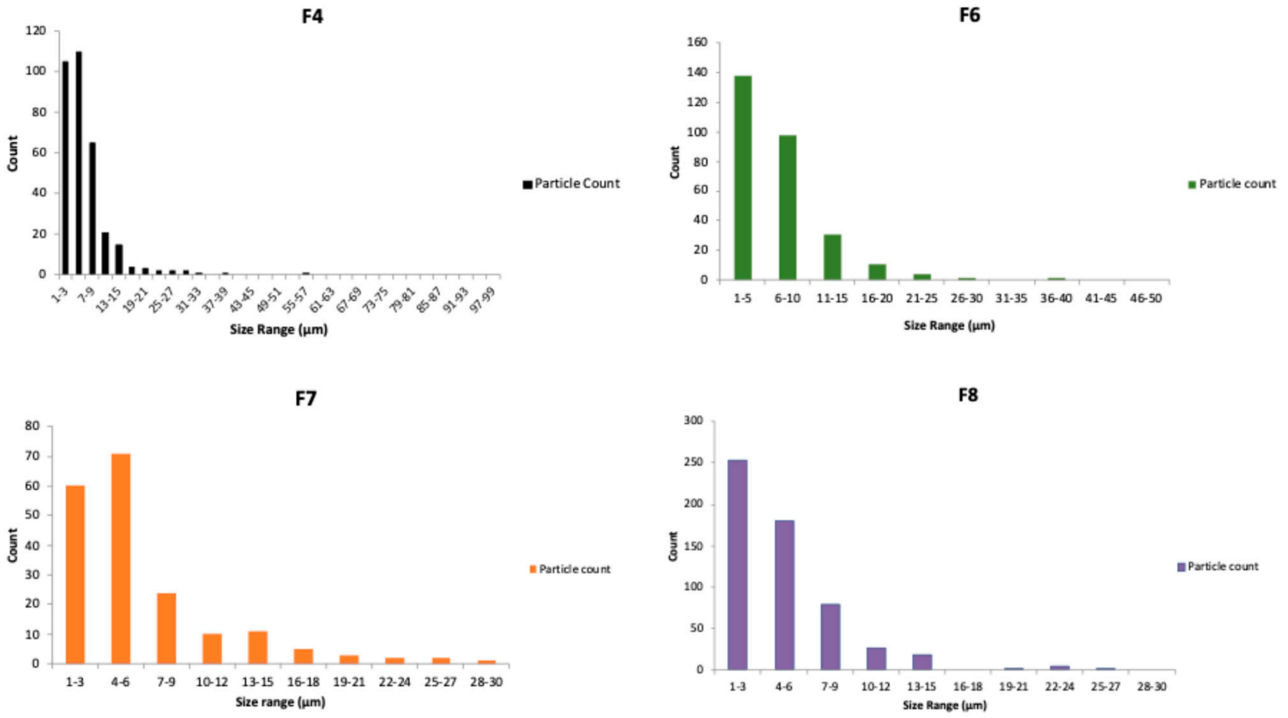

Figure S5. Distribution histograms of Que/HPβCD spray-dried (F4) and its blends (F6-F8) with MLMPs in different weight ratios (25:75, 50:50 and 75:25). The x-axis represents particle size range (µm), and the y-axis indicates particle count (n = number of particles measured).

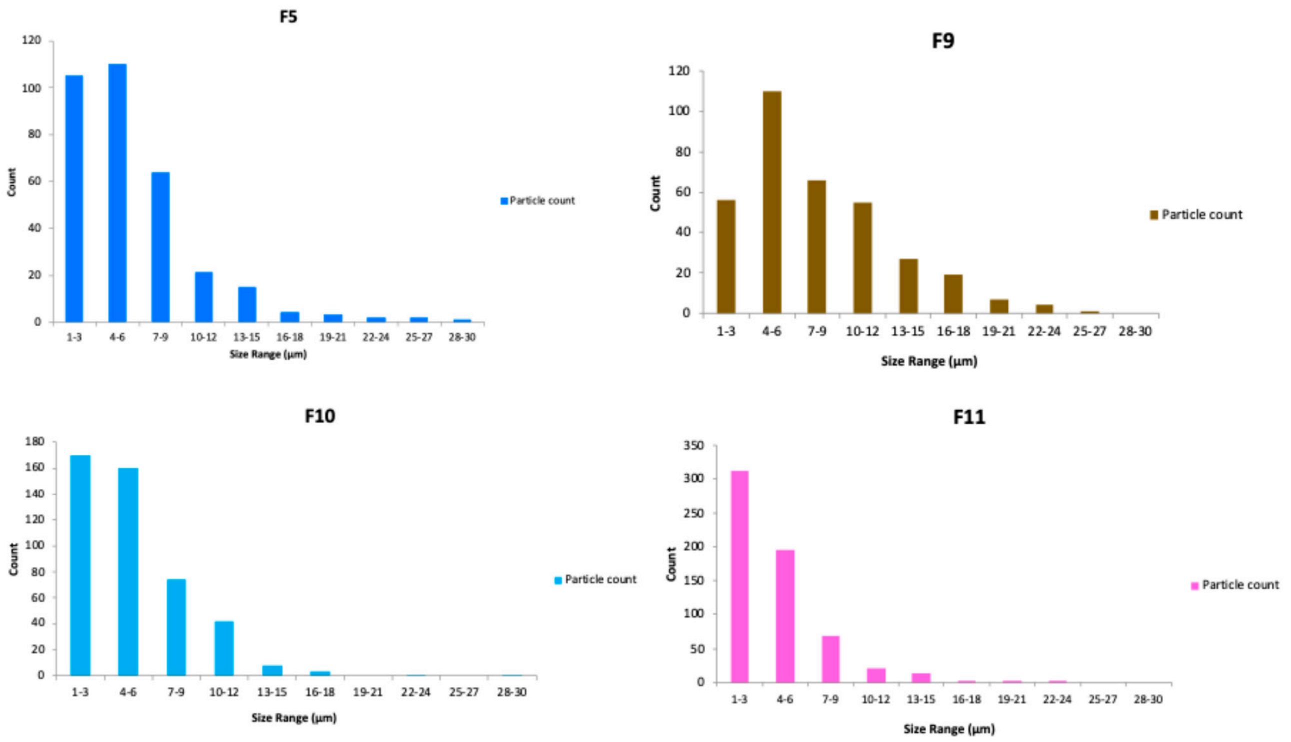

Figure S6. Distribution histograms of HPMC/HPβCD/Que spray-dried (F5).and its blends (F9-F11) with MLMPs in different weight ratios (25:75, 50:50 and 75:25). The x-axis represents particle size range (µm), and the y-axis indicates particle count (n = number of particles measured).
